# Patients’ Concerns for Hormone-Replacement Therapy for Menopause

**DOI:** 10.7759/cureus.96709

**Published:** 2025-11-12

**Authors:** Kyle R Distler, Denae Kappers, Joshua C Hollingsworth

**Affiliations:** 1 Internal Medicine, HCA Florida Orange Park Hospital, Orange Park, USA; 2 Family Medicine, HCA Florida Orange Park Hospital, Orange Park, USA; 3 Pharmacology, VCOM-Auburn, Auburn, USA

**Keywords:** hormone replacement and cancer, hormone replacement therapy, hormone therapy and menopause, menopause management, public health, risk of hormone therapy, survey study

## Abstract

Background: While most women experience significant perimenopausal symptoms, only a percentage utilize hormone-replacement therapy (HRT). Despite the publicized risks, HRT provides benefits beyond perimenopausal symptom improvement, including decreased risk of cardiovascular disease, stroke, osteoporosis, and affective disorders, and quality of life.

Methods: Subjects were recruited randomly in four clinics. The survey data is uploaded to a survey platform. Data were analyzed to identify reasons for HRT refusal, and to see what associations exist between menopause severity and use of HRT.

Results: 71 subjects in total completed the survey. 25 (35%) have never used HRT, and 46 (65%) have used it before. 17 (24%) individuals stated that concerns regarding cancer were central to decision-making on whether they opted to utilize HRT. Possible cancer risk was the most common concern (34, 48%) among the entire study population. The difference between the two groups was not significant regarding specific concerns and reasons as to why they did or did not receive this treatment for menopause. For the Menopause Rating Scale (MRS), the non-HRT group (Group 1) mean was 7.79, and the HRT group (Group 2) mean was 15.07. This difference was significant (two-tailed p = 0.0009).

Conclusions: Findings from this study show there is still a large proportion of women concerned about risks of HRT especially regarding cancer. Future studies aimed at addressing significance in HRT’s effect on osteoporosis, cardiovascular disease, and more that impact the morbidity and mortality of menopausal women could be vital to finding a unified stance on this topic.

## Introduction

Most women, at some point, experience significant symptoms of menopause (e.g., hot flashes, irritability, decreased libido, energy, sexual function, bone density, etc.), but only about 30% of eligible women utilize hormone-replacement therapy (HRT) [1]. While there are risks (e.g., deep vein thrombosis (DVT), cancer, etc.), HRT provides many potential benefits beyond perimenopausal symptom improvement, including decreased risk of cardiovascular disease, stroke, osteoporosis, and affective disorders, and improved quality of life [2-5]. It is believed that concerns regarding risks of HRT stemming from the Women’s Health Initiative (WHI) over two decades ago persist in women today [3]. The WHI findings are now considered flawed. Many of the subjects therein were over ten years post-menopause, representing a demographic from which HRT is no longer recommended [3].

More recent studies indicate that specific formulations and methods of administration are effective at relieving perimenopausal symptoms and provide additional potential benefits, including decreased risk of heart disease, improved cholesterol metabolism, neuroprotection, weight management, and more, resulting in decreased all-cause mortality when administered early after the start of menopause [3,5-10]. This area of research is important for understanding why patients are declining treatment and if these reasons are based on current understanding or risks/benefits versus previous flawed findings as potential treatment for eligible individuals could improve patient outcomes and longevity. 

This study aimed to assess: 1) perimenopausal symptom severity, 2) HRT utilization (received vs. refused), and 3) beliefs regarding HRT risks and benefits among women who have/had perimenopausal symptoms and are/were eligible for HRT.

## Materials and methods

This is a survey study on menopausal and postmenopausal patients regarding HRT, following the approval from Edward Via College of Osteopathic Medicine Institutional Review Board, Blacksburg, USA. Subjects were recruited from four clinics in Alabama and Florida (two OBGYN, one primary care, one general surgery). Patients were addressed by an investigator after being brought back to the patient room for their visit. While waiting for the provider, the investigator briefly explained the nature of the study and what was being requested of the potential subject using the study information sheet. The patient was then given time to review the information sheet and decide if they wanted to participate. If opting in, they would scan the QR code on the study handout and answer initial screening questions. If they met the inclusion criteria, the survey would continue, and the patient would complete the brief (~5 minute) survey on their phone. 

Participants completed an anonymous self-administration survey that included their Menopause Rating Scale (MRS), timeline of the start of their menopause and whether they had ever used HRT, subjects’ concerns of using HRT, and demographics (age, education, race/ethnicity) (see Appendix) [11]. Patients had to have either gone through menopause or been in menopause at time of survey to be eligible to participate. Those with a history of breast or endometrial cancer, stroke, heart attack, blood clots, or liver disease were excluded from the study. The investigator would leave the room and wait outside the patient room until the patient completed the survey. Once finished, the survey responses would be recorded under an anonymous randomized number and recorded on a data spreadsheet. Data were analyzed using sample mean and SD as well as unpaired t-test for MRS scores, and categorical variables (HRT treatment, primary health concerns, etc.) were summarized using proportions.

## Results

**Figure 1:**
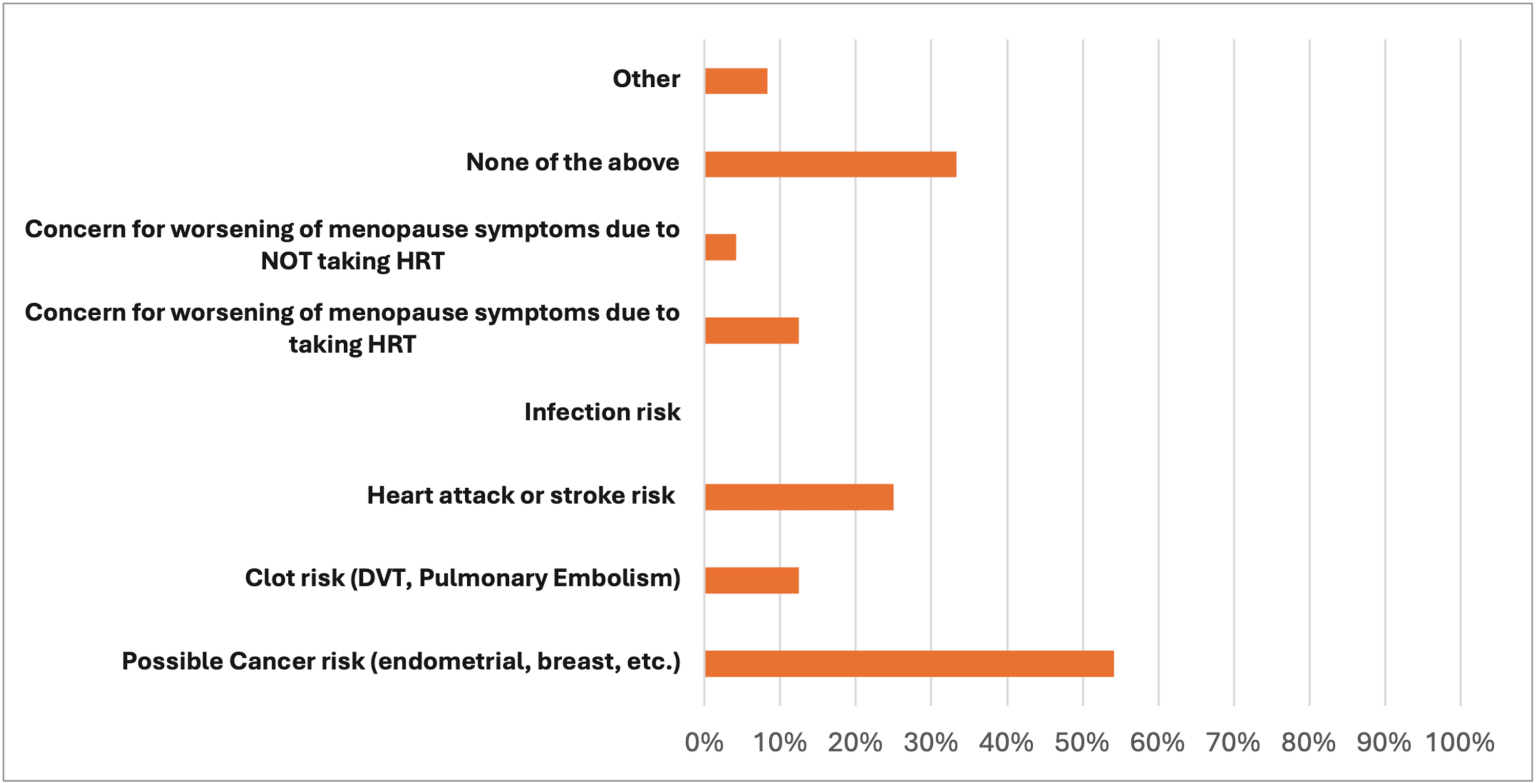
Primary health concerns when deciding to not use HRT DVT: Deep vein thrombosis; HRT: Hormone-replacement therapy

**Figure 2:**
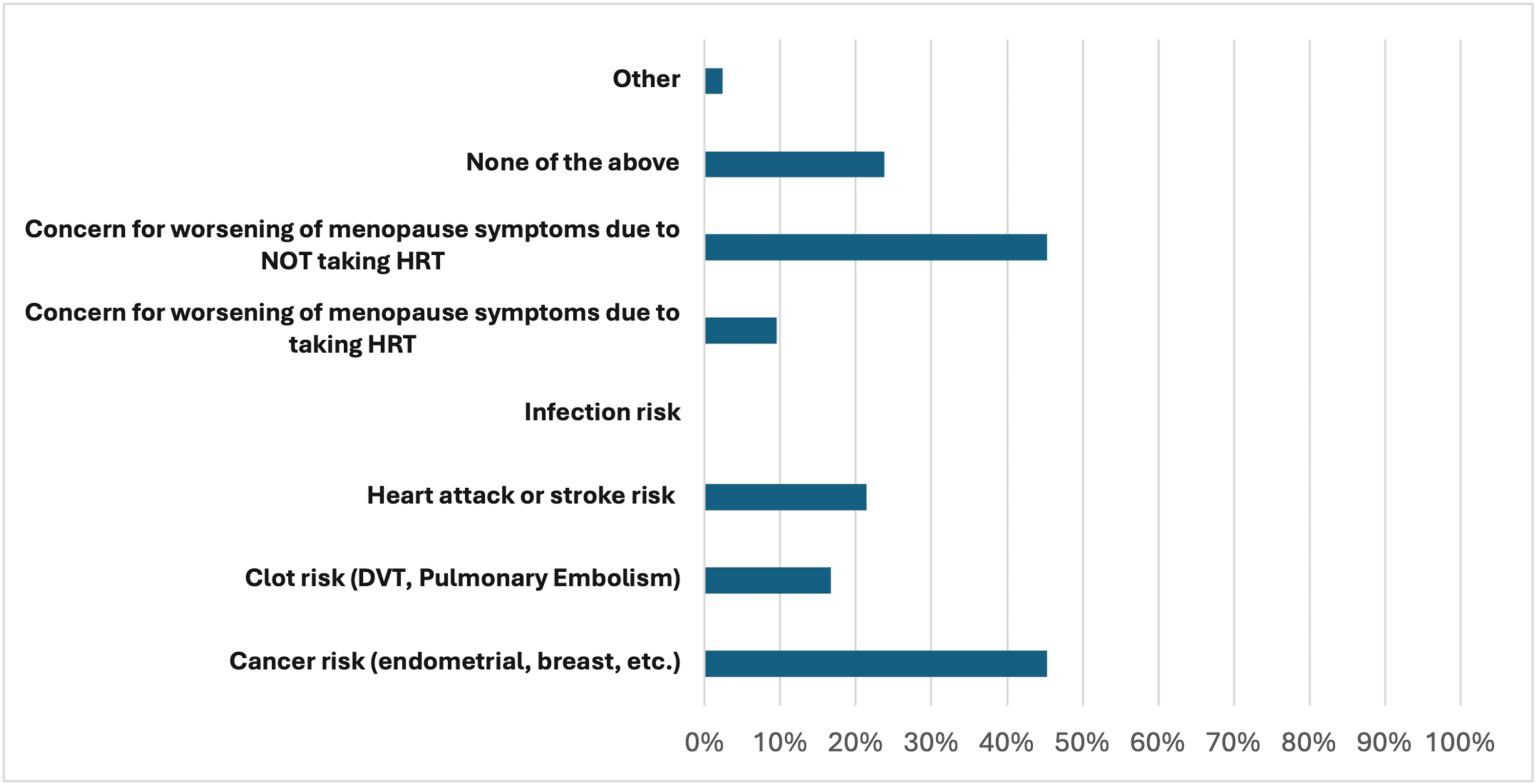
Primary health concerns when deciding to take HRT DVT: Deep vein thrombosis; HRT: Hormone-replacement therapy

71 subjects in total completed the survey. Of these, 25 (35%) have never used HRT, and 46 (65%) have used it before. For the MRS, the non-HRT group (Group 1) mean was 7.79, and the HRT group (Group 2) mean was 15.07. This difference was significant (two-tailed p = 0.0009). 17 (24%) individuals stated that concerns regarding cancer were central to decision-making on whether they opted to utilize HRT. Possible cancer risk was the most common concern (34, 48%) among the entire study population (Figures 1, 2). The difference between the two groups was not significant regarding specific concerns and reasons as to why they did or did not receive this treatment for menopause.

## Discussion

The WHI, which was conducted through the 1990s to the early 2000s, attempted to analyze risks of HRT. However, while the intent was to benefit women in providing information on options for hormone replacement for those struggling from symptoms of menopause, it inaccurately spread fear of cancer for those opting to use HRT. This is seen with the large decrease in women who use HRT for menopause-related symptoms over the last two decades, with only around 30% of women now using this treatment for menopause [1].

The findings from this anonymous survey study suggest that there is still a large proportion of women concerned about risks of HRT. While the risks of HRT of various formulations on different populations have been documented, some incorrectly, the potential benefits of HRT during menopause are still yet to be well defined for this patient population. Future studies aimed at addressing significance in HRT’s effect on osteoporosis, cardiovascular disease, and more that impact the morbidity and mortality of menopausal women could be vital to finding a unified stance on this topic. Furthermore, additional assessments analyzing quality of life between those who do and do not use HRT while suffering from severe symptoms of menopause would prove beneficial in providing patients stronger evidence for them to decide on their treatment. As risk of endometrial and breast cancer in women is high at baseline, much more research is needed to better assess the net benefit of using HRT and understanding why many providers do not motivate their patients for such treatment. Is it fear of increasing cancer risk, or is it lack knowledge of the current research used it society? If HRT really does increase the risks of endometrial and breast cancers, then why have we seen these rates of cancer continue to rise despite the decreasing use of HRT over the last 20 years [12,13].

Additional studies looking at how providers feel on HRT and risk could provide clues as to why more patients don't use it. As this large study done several years ago gained mass media and portrayed severe risk, much more education is necessary to help our patients decide on how to manage their menopause years. Additionally, specific research looking at how treating menopause with HRT during active menopause can aid in preventing metabolic syndrome, improve sleep and mental health, prevent premature osteoporosis, improve sex life, and even potentially decrease risk of myocardial infarctions in women would be ideal to provide momentum for maximizing menopause management with a more unified stance of HRT [14,15]. 

Limitations found in this study include that some patients did not comprehend how to complete the MRS portion of the online survey. Future studies with having the investigator giving the survey in person with being able to administer and ensure proper completion of the survey would improve completion rates. Furthermore, using paper handouts may improve completing the survey accurately and limit accidentally missing questions because of not understanding the online format. Lastly, it would have been best to have an equal number of subjects from each of the four clinics used. This would have made it more of a generalizable study, and future efforts should be made to use more clinics with similar numbers from each to ensure a balanced number of subjects with a more diverse group socioeconomically and education-wise. For example, a large amount of our subjects came from two of the four clinics, and many of them had bachelor's or graduate degrees. Additionally, most of our subjects were either Caucasian or African American. Beliefs and education may be diverse to what we saw here, and current beliefs of HRT and risks/benefits would be better represented if exploring a more balanced and diverse group of individuals. 

## Conclusions

Findings from this study show there is still a large proportion of women concerned about risks of HRT especially regarding cancer. While the risks of HRT of various formulations on different populations have been documented, some incorrectly, the potential benefits of HRT during menopause are still yet to be well defined for this patient population. Future studies aimed at addressing significance in HRT’s effect on osteoporosis, cardiovascular disease, and more that impact the morbidity and mortality of menopausal women could be vital to finding a unified stance on this topic. Additionally, the importance of staying up to date on current research as providers as well as the need to educate patients despite current beliefs that persist today will help them properly decide on best treatment. 

## Appendices

Patients' concerns regarding HRT for menopause 

Information Sheet for Study

The survey that you are asked to complete is part of research by Kyle Distler, DO, MS, at HCA Orange Park Hospital. 

Project: Patients‘ Concerns Regarding Hormone Replacement Therapy for Menopause 

Purpose: To see what patients believe are the primary concerns when deciding on whether or not to use hormone replacement therapy for menopause- related symptoms.

Your responses are voluntary, and the survey is anonymous. It should take you <5 minutes to complete. 

Completing and submitting the survey affirms you are ≥18 years of age and you give consent for Dr. Distler to use your responses in his research. 

Have questions about this research? Contact Dr. Distler at kyle.distler@hcahealthcare.com. 

For additional information about giving consent or your rights as a participant in this study, to discuss problems, concerns, and questions, or to offer input, please feel free to contact the Physician's Services Group Graduate Medical Education Institutional Review Board Office at 615-309-2192. This study was approved by the VCOM Institutional Review Board (VCOM IRB #2022-031) on April 26, 2024.

**Table 1:** Survey This survey has built-in logic such that the next question is dictated by subject's response in order to stratify them into two groups (HRT vs. non-HRT). DVT: Deep vein thrombosis; HRT: Hormone-replacement therapy

Number	Question
1	Do you have a history of any of the following? (Check all that apply)
	Breast or endometrial cancer
	Stroke
	Heart attack
	Blood clots
	Liver disease
	None
2	Have you already gone through or are you currently going through menopause/perimenopause?
	Yes
	No
3	What year did you begin going through menopause/perimenopause? Please provide your best guess if you are unsure.
4	Do you currently use, or have you previously used, HRT for any reason(s) related to menopause/perimenopause? HRT includes the use of an estrogen with or without a progestin, taken in any dosage form (tablet, patch, gel, spray, injection, ring, or cream).
	Yes
	No
5	What primary health concern, if any, stopped you from taking HRT?
6	Which of the following health concerns influenced your decision to not take HRT? Check all that apply.
	Possible cancer risk (endometrial, breast, etc.)
	Clot risk (DVT, pulmonary embolism)
	Heart attack or stroke risk
	Infection risk
	Concern for worsening of menopause symptoms due to taking HRT
	Concern for worsening of menopause symptoms due to NOT taking HRT
	None of the above
	Other
7	What year did you begin taking HRT? Please provide your best guess if you are unsure.
8	What primary health concern, if any, almost stopped you from taking HRT?
9	What were your primary concerns when deciding to take HRT? (Check all that apply)
	Cancer risk (endometrial, breast, etc.)
	Clot risk (DVT, pulmonary embolism)
	Heart attack or stroke risk
	Infection risk
	Concern for worsening of menopause symptoms due to taking HRT
	Concern for worsening of menopause symptoms due to NOT taking HRT
	None of the above
	Other
10	(For the next 11 questions): Thinking about your time going through menopause/perimenopause, please select "None" for each symptom below that you did not experience, and select the overall severity (Mild, Moderate, Severe, or Extremely Severe) for each symptom below that you did experience.
	1. Hot flashes, sweating (episodes of sweating)
	2. Heart discomfort (unusual awareness of heartbeat, heart skipping, heart racing, tightness)
	3. Sleep problems (difficulty in falling asleep, difficulty in sleeping through the night, waking up early)
	4. Depressive mood (feeling down, sad, on the verge of tears, lack of drive, mood swings)
	5. Irritability (feeling nervous, inner tension, feeling aggressive)
	6. Anxiety (inner restlessness, feeling panicky)
	7. Physical and mental exhaustion (general decrease in performance, impaired memory, decrease in concentration, forgetfulness)
	8. Sexual problems (change in sexual desire, in sexual activity and satisfaction)
	9. Bladder problems (difficulty in urinating, increased need to urinate, bladder incontinence)
	10. Dryness of vagina (sensation of dryness or burning in the vagina, difficulty with sexual intercourse)
	11. Joint and muscular discomfort (pain in the joints, rheumatoid complaints)
11	(For the next 11 questions): Thinking about your time going through menopause/perimenopause before you started HRT, please select "None" for each symptom below that you did not experience, and select the overall severity (Mild, Moderate, Severe, or Extremely Severe) for each symptom below that you did experience.
	1. Hot flashes, sweating (episodes of sweating)
	2. Heart discomfort (unusual awareness of heartbeat, heart skipping, heart racing, tightness)
	3. Sleep problems (difficulty in falling asleep, difficulty in sleeping through the night, waking up early)
	4. Depressive mood (feeling down, sad, on the verge of tears, lack of drive, mood swings)
	5. Irritability (feeling nervous, inner tension, feeling aggressive)
	6. Anxiety (inner restlessness, feeling panicky)
	7. Physical and mental exhaustion (general decrease in performance, impaired memory, decrease in concentration, forgetfulness)
	8. Sexual problems (change in sexual desire, in sexual activity and satisfaction)
	9. Bladder problems (difficulty in urinating, increased need to urinate, bladder incontinence)
	10. Dryness of vagina (sensation of dryness or burning in the vagina, difficulty with sexual intercourse)
	11. Joint and muscular discomfort (pain in the joints, rheumatoid complaints)
12	Please enter your current age in years.
13	Please select the race/ethnicity that best defines you. Select all that apply.
	Hispanic or Latino or Spanish Origin of any race
	American Indian or Alaskan Native
	Asian
	Native Hawaiian or other Pacific Islander
	Black or African American
	White
	Two or more races
14	Please select the highest level of education that you have completed.
	Nursery school to 8^th^ grade
	Some high school, no diploma
	High school graduate, diploma or equivalent
	Some college credit, no degree
	Trad/technical/vocational training
	Associate degree
	Bachelor’s degree
	Master’s degree
	Professional degree
	Doctorate degree
